# Iodine Concentration in Breastmilk and Urine among Lactating Women of Bhaktapur, Nepal

**DOI:** 10.3390/nu8050255

**Published:** 2016-04-28

**Authors:** Sigrun Henjum, Marian Kjellevold, Manjeswori Ulak, Ram K. Chandyo, Prakash S. Shrestha, Livar Frøyland, Emmerentia E. Strydom, Muhammad A. Dhansay, Tor A. Strand

**Affiliations:** 1 Oslo and Akershus University College of Applied Sciences, P.O. Box 4, St. Olavs plass, Oslo 0130, Norway; 2 National Institute of Nutrition and Seafood Research (NIFES), Bergen 5817, Norway; Marian.Kjellevold@nifes.no (M.K.); Livar.Froyland@nifes.no (L.F.); 3 Department of Child Health, Institute of Medicine, Tribhuvan University ,Maharajgunj, P.O. Box 1524, Kathmandu 44600, Nepal; manjeswori@gmail.com (M.U.); prakashsunder@hotmail.com (P.S.S.); 4 Centre for International Health, University of Bergen, P.O. Box 7800, Bergen 5020, Norway; ram.chandyo@uib.no; 5 Burden of Disease Research Unit, South African Medical Research Council, P.O. Box 19070, Tygerberg 7505, South Africa; Emmerentia.Strydom@mrc.ac.za (E.E.S.); ali.dhansay@mrc.ac.za (M.A.D.); 6 Division of Human Nutrition and Department of Paediatrics and Child Health, Faculty of Medicine and Health Sciences, Stellenbosch University, Francie van Zijl Drive, Tygerberg Hospital, Tygerberg 7505, South Africa; 7 Department of Research, Innlandet Hospital Trust, Lillehammer 2629, Norway; tor.strand@uib.no

**Keywords:** iodine concentration, breastmilk, lactating women, Nepal, urinary iodine, excess iodine

## Abstract

Adequate iodine concentration in breastmilk (BMIC) is essential for optimal neonatal thyroid hormone synthesis and neurological development in breastfed infants. For many decades, iodine deficiency has been a public health problem in Nepal. However, recently, excessive iodine intakes among Nepali infants have been reported. This study aimed to measure BMIC and urinary iodine concentration (UIC) among lactating women in a peri-urban area of Nepal. Iodine concentration was measured in spot urine (*n* = 485) and breastmilk samples (*n* = 291) of 500 randomly selected lactating women. The median (p25, p75) BMIC and median UIC were 250 (130, 370) µg/L and 230 (135–377) µg/L, respectively. Around 82% had BMIC > 100 µg/L, 61% had BMIC > 200 µg/L and 81% had UIC > 100 µg/L, 37% had >300 µg/L and 20% had >500 µg/L. In multiple linear regression models, time since birth (β 3.0, 95% CI (0.2, 5.0)) and UIC (β 1.0, 95% CI (0.1, 2.0)) were associated with BMIC, explaining 26% of the variance. A large proportion of the women had adequate BMIC and UIC; however, a subset had high iodine concentrations. These findings emphasize the importance of carefully monitoring iodine intake to minimize the risk of iodine excess and subsequently preventing transient iodine-induced hypothyroidism in breastfed infants.

## 1. Introduction

Iodine deficiency disorders (IDD) are a major public health problem worldwide in all age groups, but infants, schoolchildren, and pregnant and lactating women are most vulnerable [1]. During pregnancy and lactation, the fetus and infants are sensitive to maternal iodine intake and an adequate iodine concentration in breastmilk (BMIC) is essential for optimal growth and neurological development [2]. Exclusively breastfed infants depend entirely on their mother’s milk iodine content for thyroid hormone synthesis since they, unlike adults, lack significant thyroxine stores [3]. In order to meet the infants’ iodine requirements, the World Health Organization (WHO) recommends an iodine intake of 250 µg/day for lactating and pregnant women [1]. The median urinary iodine excretion is the recommended biomarker to monitor daily iodine intake in a given population, and in non-lactating women, approximately 90% of the ingested iodine is excreted through urine [1,4]. According to Laurberg, in lactating women who consume the recommended 250 µg iodine per day, around 40%–45% of the iodine intake is transported into breastmilk by the sodium iodide transporter (NIS) and urinary iodine excretion is consequently lower [5].

The Himalayan region in Nepal is one of the most iodine-deficient areas in the world [6] and, for many decades, iodine deficiency disorders have been a public health problem [7]. Recently, excessive iodine intake among Nepali infants and school children have been reported [8,9,10,11,12]. One study explained the excessive iodine intake by use of over-iodized salt; however, another study found salt samples within acceptable levels. The risk of developing thyroid disease increases with both low and high iodine intake [1]. Infants are especially vulnerable to developing subclinical hypothyroidism from excessive iodine intake [10,13,14]. Despite the importance of iodine to infant growth and development, there are only a few studies related to the iodine content of human milk in the scientific literature. This study aimed to measure BMIC and urinary iodine concentration (UIC) in a large random sample of lactating women in a peri-urban area of Nepal.

## 2. Materials and Methods

### 2.1. Subjects

A cross-sectional survey on micronutrient intake and status was conducted during 2008–2009 in 500 healthy lactating women (15–45 years old) and their breastfed infants (2–12 months) in Bhaktapur municipality in Nepal. The study purpose of this paper was to evaluate the iodine status of the lactating mothers. The selection criteria, the field procedures and a flow chart of the recruitment of the study subjects have been published elsewhere [15]. The sample size was chosen based on the assumption that the prevalence of dietary deficiency of key micronutrients would be 25%. A total of 450 lactating women were required to detect this prevalence with an absolute precision of 4%, *i.e.*, with a 95% confidence interval from 21% to 29%. Assuming incomplete sampling from approximately 10% of these women, we calculated a final desired sample size of 500 women. We used a two-stage cluster sampling procedure whereby 66 neighborhoods (“Toles”) were randomly selected as the primary sampling unit from a total of 160. We listed all women living in these toles and randomly selected the 500 women [16]. The inclusion criteria for the study were that the women had no ongoing infection (clinically assessed), resided in the selected clusters, were willing to provide their household information, and consented to participate. Because of acute blood loss and physiological and hormonal changes during delivery that usually last up to two months afterward [17], we enrolled lactating women two months after delivery. In total, 582 women were approached for enrollment and 500 were included in the study; 485 provided a urine sample, and 291 of the same women donated a sample of breastmilk. Of the breastmilk samples, 117 were collected up to 6 months postpartum and 174 from 7 to 12 months postpartum.

### 2.2. Ethics

All women provided written informed consent before the start of the study. Ethical clearance was obtained from the institutional review boards at the Institute of Medicine at Tribhuvan University in Kathmandu, Nepal.

### 2.3. Urine and Breastmilk Collection

Approximately 5 mL spot urine samples and 5 mL breastmilk (non-fasting samples) were collected from each woman, and aliquots were stored at −20 °C until analyzed. Vacuette Urine System with transfer devices (Vacuette, Krensmünster, Austria) were used for sampling and storage of the urine samples. For the breastmilk samples, Sarstedt 20 mL plastic tubes were used. Women collected breast-milk by manual expression and the breastmilk was collected on the same day and close in time to the collection of the urine samples.

### 2.4. Analytical Procedures

The iodine concentrations in the urine (UIC) samples were measured at the Nutritional Intervention Research Unit of the Medical Research Council in Cape Town, South Africa by manual digestion with ammonium persulfate followed by measuring the Sandell-Kolthoff reaction spectrophotometrically using 96-well plates and an absorbance microplate reader at 405 nm [18,19]. 

The analytical quality of method was validated by participating regularly in an external quality assurance programme namely EQUIP of CDC, Atlanta, USA (bias 1.9%, 1.5%, 2.4% at UI concentrations of 105, 263 and 494 ug/L). The inter assay variations (% CVs) of in-house urine controls were 8.0%, 7.5%, 3.8% and 7.0% at concentration of 85, 115, 225, 294 µg/L of, while the % recovery of iodine added to aqueous solution were 98% and 97% at 160 and 240 µg/L (RSD % were 3.3 and 2.6).

The BMIC samples (*n* = 291) were determined by inductive coupled plasma-mass spectrometry (ICP-MS), as described in Julshamn *et al*. (2001) [20], at the National Institute of Nutrition and Seafood Research (NIFES) in Norway. This method is accredited for food, including milk, and validated for concentrations between 0.04 and 5.0 mg/kg of dry weight. The analytical quality of the method and systematic errors were controlled by using the certified European Reference Material (ERM 150) (skimmed milk powder). The intra-assay variation (% CV) of ERM-150 was 3.3% (*n* = 20).

### 2.5. Definitions

A median UIC above 100 µg/L in lactating women is regarded as sufficient, according to the WHO recommendations [1]. An exact cut-off for BMIC has not been specified; however, breastmilk with iodine concentration above 75 µg/L may be considered as an index of sufficient iodine intake [2]. Full-term infants need 15 μg iodine/kg daily to maintain positive iodine balance, which would equate to a BMIC of 100 to 200 μg/L [21,22]. The estimated iodine intake among infants 0–6 months was calculated based on a mean estimated consumption of breast-milk by 0–6-month-old infants of approximately 0.8 L/day [23,24]. BMI was calculated as weight/(height)^2^ (kg/m^2^). BMI < 18.5 kg/m^2^ was considered as underweight, 18.5 kg/m^2^ < BMI ≥ 25 kg/m^2^ as normal weight and BMI ≥ 25 kg/m^2^ as overweight [25].

### 2.6. Statistical Analysis

The data were analyzed by using SPSS version 22 (SPSS, Inc., New York, NY, USA) and STATA version 14 (Stata Corporation, Inc., College Station, TX, USA). Parametric data are presented as mean (SD) and non-parametric data as median (p25, p75). Two-tailed tests with a 5% significance level were used for all analyses. Spearman’s correlation analysis was performed to investigate the association between time since birth and the UIC and BMIC. The UIC and BMIC were used as dependent variables in multiple linear regression analyses. Because of skewed distributions, these dependent variables were log (base 2)-transformed. A theory-based approach was used to select candidate variables for inclusion in the model. Thus, the following variables known to influence UIC and BMIC, and selected socioeconomic variables, were included in the initial crude models: the mother’s age, number of children, the child’s age, exclusive breastfeeding, frequency of breastfeeding per day, child’s weight for age < −2Z score, length for age < −2Z score, length for weight < −2Z score, housing type (rented or owned), land ownership, type of family, and season of the year (spring (April–June); summer (July–September); autumn (October–December); and winter (January–March)). All covariates showing a linear association (*p* < 0.10) in the crude regression models were included in a preliminary multiple regression model. Excluded variables were reintroduced and those who were still significantly associated in this model (*p* < 0.10) were retained in the final model. Analysis of the residuals was performed in order to examine the fit of the model. The dose-response graphs were constructed using kernel-weighted local polynomial regression in Stata 14.

## 3. Results

Table 1 presents the background data on the lactating Nepalese women. The mean (SD) ages of the women and children were 25.7 (4.1) years and 6.8 (3.0) months, respectively. Approximately 42% of the women had two children, and 18% had three or more children. A total of 51% of the women had exclusively breastfed up to 3 months and 18% up to 6 months. The mean (SD) frequency of breastfeeding (daytime) was 11.1 (3.3) from 0 to 3 months, 10.3 (3.6) from 4 to 6 months, 9.1 (3.1) from 7 to 9 months and 8.8 (3.3) from 10 to 12 months.

**Figure 1:**
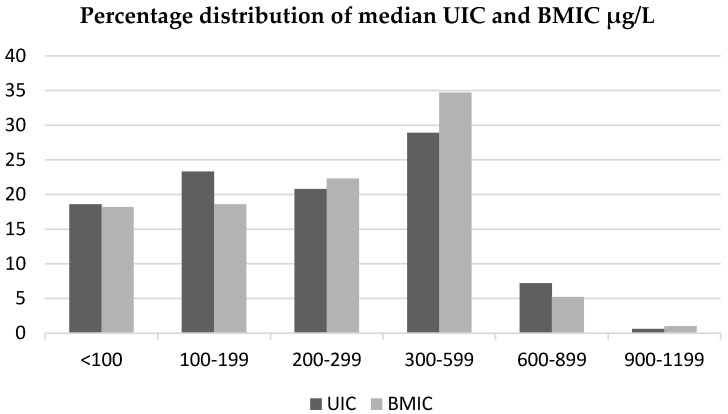
Percentage distribution of median urinary iodine concentration and breastmilk iodine concentration (µg/L).

Figure 1 shows the percentage distribution of the median BMIC and UIC among the women. The median (p25, p75) BMIC was 250 (130, 370) µg/L, and 90% of the women had BMIC > 75 µg/L, 82% had BMIC > 100 µg/L and 61% had BMIC > 200 µg/L. The median (p25, p75) UIC was 230 (135–377) µg/L; 81% had a UIC > 100 µg/L, 37% had UIC > 300 µg/L and 20% had UIC > 500 µg/L. Only 6% had UIC less than 50 µg/L. No seasonal differences were found in UIC. The estimated iodine intake of infants 0–6 months, based on a median BMIC of 250 µg/L and a mean estimated consumption of breastmilk of 0.8 L/day, was 200 µg/day. The mean (SD) estimated total iodine excretion among the lactating women via breastmilk and urine was 537 (323) µg/day.

**Figure 2:**
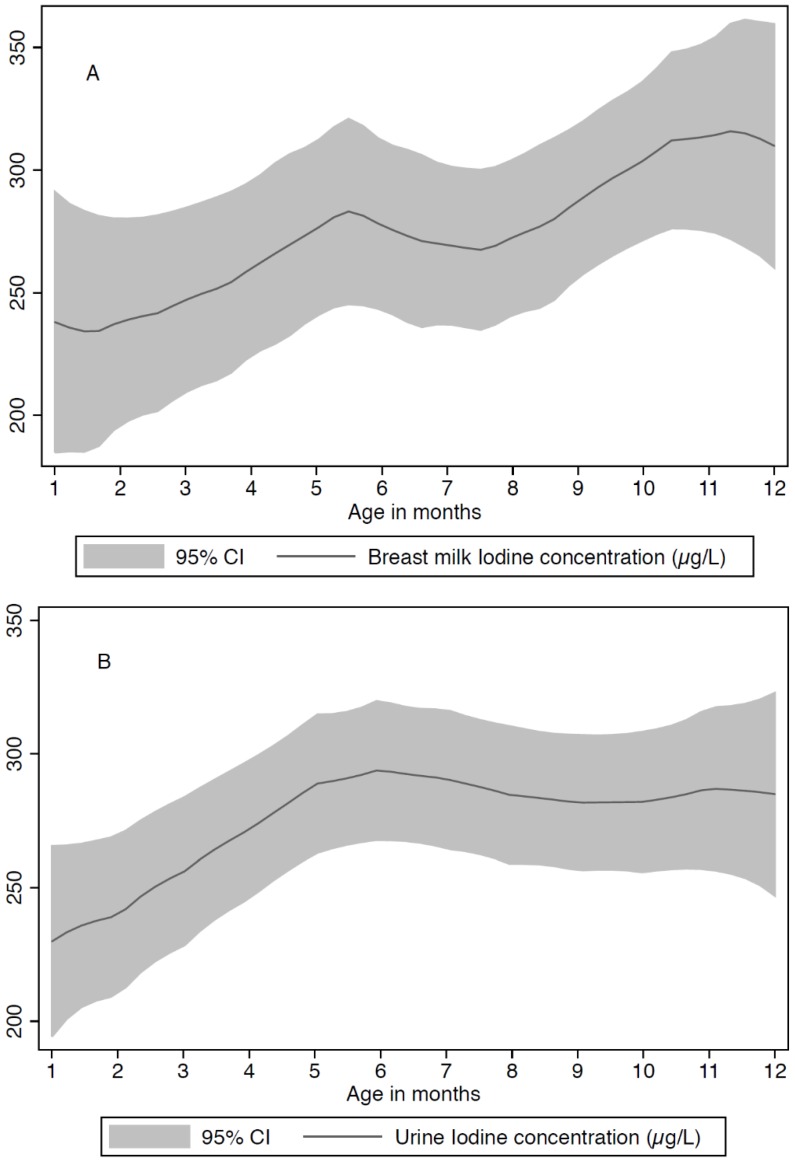
Association of breastmilk iodine concentration (BMIC) (**A**) and maternal urinary iodine concentration (UIC) (**B**) and time since birth (age of the child). The shaded areas represent the 95% CI of the smooth regression line.

The association of BMIC and UIC and time since birth is presented in Figure 2A,B, (Supplementary Table S1). The median BMIC increases with time since birth, peaking at around 11–12 months. The UIC increases up to 5–6 months since birth and then levels out.

**Figure 3:**
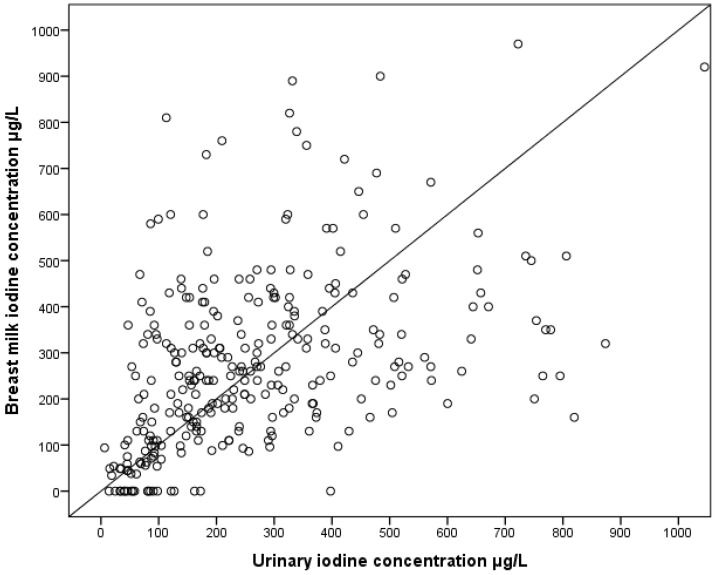
Correlation between UIC and BMIC, *p* < 0.001, *r*^2^ = 0.20.

A weak but significant correlation between UIC and BMIC was found, *r*^2^ = 0.2 and *p* < 0.001 (Figure 3).

In multiple linear regression models (Table 2), time since birth (β 3.0, 95% CI (0.2, 5.0)) and UIC (β 1.0, 95% CI (0.1, 2.0)) were associated with BMIC, explaining 26% of the variance.

## 4. Discussion

In this study of a representative sample of 500 lactating women in peri-urban Nepal, a large proportion of the women had an adequate BMIC and UIC; moreover, a subset had high iodine concentrations.

A review on breastmilk iodine reported a wide range of iodine concentrations (13–155 µg/L) among women living in areas with varying levels of iodine intake [2]. In countries with sufficient iodine intakes, the BMIC have been reported to be between 150 and 180 μg/L [26,27,28]. A number of international researchers and WHO have recommended that BMIC is optimal at concentrations of 100–200 μg/L to ensure normal development in infants [27,28,29]. In our study, 61% of the women had BMIC > 200 µg/L. A recent study from Eastern Nepal found adequate UIC (175 µg/L) in lactating women and a median BMIC of 130 µg/L [30]. The median BMIC in our study was 250 µg/L [23,24] and the estimated iodine intake of the infants (0–6 months) was 200 µg/day. This iodine intake is above the WHO’s recommended maximum of 180 µg/day for infants <2 years old [31] and the US Institute of Medicine’s tolerable upper limit of 200 µg/day for 1–3-year-old children [32]. In our study, 50% of the infants were exclusively breastfed at 3 months, and these children are at risk of consuming excessive iodine intakes through breastmilk. In older children who are not exclusively breastfed, the total iodine intake also depends on the iodine content of the complementary food. However, the most common complementary food in the area is Lito—a local semisolid porridge [33], which is not fortified with iodine or other micronutrients. Fetus and newly born are high-risk groups for excessive iodine exposure since their thyroid gland is less able to adapt to high iodine intake and, as a result, may develop subclinical hypothyroidism [34,35]. Given that the brain is growing rapidly during infancy, iodine excess and thyroid hormone disturbances could permanently affect their neurodevelopment [36].

The median UIC (230 µg/L) in this population was above the WHO recommendation of an UIC > 100 µg/L [1]; and 37% had UIC > 300 µg/L and 20% had UIC > 500 µg/L. Excessive iodine intake in lactating women may induce subclinical hypothyroidism [14]. Increased thyroid dysfunction among Nepali women has been reported in the Kathmandu region [37]. No seasonal variation in UIC was found, contrary to another study conducted in Nepal [38].

The sampling of breastmilk for iodine measurements may be affected by physiological fluctuations of iodine content [39]. The data from our study showed that median BMIC gradually increased with time since birth up to 11–12 months. In small observational studies, breastmilk iodine levels were reported to increase during the first postpartum month [40], decrease during the first 6 postpartum months [41], and vary from day to day [42]. A decline in BMIC in the postpartum period may be unique to women with suboptimal iodine status and should be confirmed in a longitudinal study of breastfeeding women with adequate iodine status [41]. In multiple linear regression analyses, we found a positive association between BMIC and UIC. According to United States National Health and Nutrition Examination Survey (NHANES), in areas with iodine sufficiency, BMIC correlates with UIC [43]. This was also found in a study of lactating mothers in Bangladesh [44]. In China, in an area of iodine excess, Liu *et al*., found that BMIC was positively correlated with UIC in lactating women [14].

Since 1973, salt iodization has constituted the major defense against iodine deficiency in Nepal [38]. Data from the National Demographic Household Survey (2011) showed that eight of ten households in Nepal have adequately iodized salt (15+ ppm) and adequately iodized salt is more commonly found in urban (94%) than rural households (78%) [7]. In Nepal, there are regional differences in iodine status, with a higher prevalence of iodine deficiency disorders in the far mountainous and hilly regions than in the Terai region (eastern plains) [45,46,47,48]. A recent study from the Terai region reported that 34% of school-aged children (SAC) had excessive UIC [8]. Another study found that 40% of SAC in the hills and eastern plains had excessive UIC, together with a 30% prevalence of subclinical hypothyroidism [9]. An excessive iodine intake and subclinical hypothyroidism, together with elevated thyroglobulin levels, have also been found among 6–24-month-old infants in the eastern region of Nepal [10]. Although the current Nepalese legislation stipulates a level of iodine at 50 ppm (85 ppm of potassium iodate) at the production level, 30 ppm at retail level and 15 ppm at the household level, the excessive iodine intake in this study was explained by use of over-iodized salt (median salt iodine concentration of 89 ppm) [10]. A study found that 50% of SAC in the hills and plains had excessive iodine intakes and the salt samples from their homes were within acceptable levels [11]. The authors suggested that frequent consumption of uncooked instant noodles and flavor sachets with high iodine levels could be part of the explanation [11]. The dietary intake of the lactating women in our study was measured by three repetitive 24 h recalls during one year and is presented elsewhere [49], and uncooked instant noodles was not frequently consumed. Additionally, the intake of iodine-rich foods, such as fish and seafood, was scarce. We therefore assumed that the most important source of iodine among the lactation women was iodized salt.

To the best of our knowledge, this is the first study that has published data on BMIC from this peri-urban area of Nepal. The strength of the study lies in the high response rate from a large, representative and random sample of lactating women, as well as the large number of urine and breastmilk samples obtained. A limitation of this study was that we did not have information on UIC in the infants, as well as lack of information about dietary sources of iodine and the iodine level in salt and water.

## 5. Conclusions

In conclusion, a large proportion of the lactating women had adequate BMIC and UIC; however, a subset had high iodine concentrations. This is of special concern since the newborn thyroid is less able to adapt to high iodine intake from breastmilk and this may result in subclinical hypothyroidism. In iodized salt programs, regular and careful monitoring and surveillance of UIC and salt iodine concentration are warranted, not only to control iodine deficiency but also to minimize the risk of iodine excess.

We suggest that further investigation in this peri-urban area of Nepal collect household salt samples and determine the level of iodine in the salt, as well as determine the UIC in infants.

## Figures and Tables

**Table 1:** Background data on the lactating Nepali women (*n* = 485).

Age of women, years (mean, SD)	25.7 ± 4.1
Age of child, months (mean, SD)	6.8 ± 3.0
Home delivery, %	10.1
Parity	
One, %	40.9
Two, %	41.5
Three or more, %	17.6
Exclusive breastfeeding ^1^ 3 months, %	50.2
Exclusive breastfeeding 6 months, %	16.4
Frequency of breastfeeding (mean, SD)	9.7 ± 3.4
Body Mass Index (kg/m^2^) (mean, SD)	22.4 ± 3.1
<18.5, %	4.7
18.5–25, %	78.7
>25, %	16.6

^1^ Exclusive breastfeeding defined as a child only consuming breastmilk and no other fluid or food except medicine. Prevalence calculated for those who had passed the relevant age.

**Table 2:** Determinants of BMIC among lactating women (*n* = 291).

Dependent Variables ^a^	Predictor Variables	Unadjusted Beta Coefficients ^d^ (95% CI)	*p*	Adjusted Beta Coefficients ^d^ (95% CI) ^b^	*p*	Stand Beta
BMIC, µg/L	Constant			5.58 (4.58, 6.59)		
	UIC, µg/L	0.2 (0.1, 0.2)		1.0 (0.1, 2.0)	<0.01	0.41
	Time since birth ^c^	4.0 (1.0, 6.0)	<0.001	3.0 (0.2, 5.0)	0.03	0.12
*R*^2^					0.26	

^a^ BMIC log (2) transformed; ^b^ BMIC adjusted for: Exclusive breastfeeding; ^c^ Time since birth in months; ^d^ The beta coefficients are multiplied with 100.

## Supplementary Materials

The following are available online at http://www.mdpi.com/2072-6643/8/5/255/s1, Table S1: Iodine concentration in urine (*n* = 485) and breastmilk (*n* = 291) among lactating women in Nepal.

## Abbreviations

The following abbreviations are used in this manuscript:

BMIC	Breastmilk iodine concentration
ERM	European Reference Material
IDD	Iodine deficiency disorders
ICP-MS	Inductive coupled plasma-mass spectrometry
SAC	School aged children
UIC	Urinary iodine concentration
