# Supplementary material for: Iodine Concentration in Breastmilk and Urine among Lactating Women of Bhaktapur, Nepal
Source: Nutrients. 2016 Apr 28;8(5):255. doi: 10.3390/nu8050255 (PMC4882668; doi:10.3390/nu8050255)
Supplement: Supplementary file 1 [file nutrients-08-00255-s001.docx]

Supplementary Materials: Iodine Concentration in Breast Milk and Urine among Lactating Women of Bhaktapur, Nepal

Sigrun Henjum, Marian Kjellevold, Manjeswori Ulak, Ram K. Chandyo, Prakash S. Shrestha, Livar Frøyland, Emmerentia E. Strydom, Muhammad A. Dhansay and Tor A. Strand

**Table S1.** Iodine concentration in urine (*n* = 485) and breastmilk (*n* = 291) among lactating women
in Nepal.

| **Age Months** | **Iodine Urine** | **Iodine Breastmilk** |
| --- | --- | --- |
| 11 | 722 | 970 |
| 6 | 1045 | 920 |
| 9 | 484 | 900 |
| 4 | 332 | 890 |
| 10 | 327 | 820 |
| 11 | 113 | 810 |
| 2 | 339 | 780 |
| 6 | 210 | 760 |
| 11 | 356 | 750 |
| 9 | 182 | 730 |
| 10 | 422 | 720 |
| 10 | 477 | 690 |
| 6 | 571 | 670 |
| 7 | 446 | 650 |
| 10 | 323 | 600 |
| 8 | 454 | 600 |
| 7 | 121 | 600 |
| 5 | 177 | 600 |
| 11 | 320 | 590 |
| 8 | 99 | 590 |
| 4 | 86 | 580 |
| 10 | 391 | 570 |
| 9 | 402 | 570 |
| 5 | 510 | 570 |
| 10 | 653 | 560 |
| 9 | 185 | 520 |
| 7 | 415 | 520 |
| 7 | 736 | 510 |
| 4 | 806 | 510 |
| 7 | 745 | 500 |
| 10 | 652 | 480 |
| 10 | 328 | 480 |
| 3 | 295 | 480 |
| 2 | 270 | 480 |
| 8 | 67 | 470 |
| 5 | 527 | 470 |
| 2 | 358 | 470 |
| 12 | 522 | 460 |
| 9 | 258 | 460 |
| 8 | 239 | 460 |
| 7 | 139 | 460 |
| 5 | 196 | 460 |
| 8 | 406 | 450 |
| 11 | 294 | 440 |
| 7 | 176 | 440 |
| 6 | 395 | 440 |
| 4 | 140 | 440 |
| 12 | 118 | 430 |
| 11 | 299 | 430 |
| 10 | 658 | 430 |
| 10 | 436 | 430 |
| 6 | 405 | 430 |
| 9 | 256 | 420 |
| 8 | 327 | 420 |
| 8 | 153 | 420 |
| 7 | 148 | 420 |
| 6 | 507 | 420 |
| 6 | 302 | 420 |
| 6 | 299 | 420 |
| 11 | 272 | 410 |
| 11 | 176 | 410 |
| 10 | 71 | 410 |
| 3 | 180 | 410 |
| 11 | 671 | 400 |
| 10 | 324 | 400 |
| 2 | 644 | 400 |
| 9 | 85 | 390 |
| 6 | 195 | 390 |
| 3 | 383 | 390 |
| 3 | 335 | 390 |
| 8 | 335 | 380 |
| 8 | 202 | 380 |
| 11 | 754 | 370 |
| 11 | 237 | 370 |
| 10 | 327 | 360 |
| 10 | 321 | 360 |
| 10 | 179 | 360 |
| 10 | 153 | 360 |
| 6 | 47 | 360 |
| 5 | 92 | 360 |
| 3 | 296 | 360 |
| 11 | 770 | 350 |
| 11 | 388 | 350 |
| 10 | 779 | 350 |
| 8 | 472 | 350 |
| 10 | 95 | 340 |
| 8 | 483 | 340 |
| 4 | 520 | 340 |
| 4 | 243 | 340 |
| 3 | 332 | 340 |
| 2 | 81 | 340 |
| 11 | 358 | 330 |
| 9 | 641 | 330 |
| 9 | 341 | 330 |
| 8 | 97 | 330 |
| 7 | 191 | 330 |
| 6 | 295 | 330 |
| 11 | 172 | 320 |
| 11 | 113 | 320 |
| 9 | 481 | 320 |
| 9 | 73 | 320 |
| 5 | 389 | 320 |
| 3 | 874 | 320 |
| 3 | 273 | 320 |
| 12 | 205 | 310 |
| 11 | 206 | 310 |
| 10 | 121 | 310 |
| 9 | 250 | 310 |
| 8 | 177 | 310 |
| 6 | 356 | 310 |
| 3 | 406 | 310 |
| 2 | 159 | 310 |
| 11 | 196 | 300 |
| 11 | 128 | 300 |
| 10 | 140 | 300 |
| 9 | 183 | 300 |
| 6 | 445 | 300 |
| 5 | 182 | 300 |
| 4 | 270 | 300 |
| 8 | 209 | 290 |
| 5 | 560 | 290 |
| 4 | 220 | 290 |
| 11 | 516 | 280 |
| 9 | 267 | 280 |
| 7 | 436 | 280 |
| 5 | 131 | 280 |
| 4 | 130 | 280 |
| 11 | 243 | 270 |
| 9 | 571 | 270 |
| 9 | 532 | 270 |
| 9 | 320 | 270 |
| 9 | 53 | 270 |
| 8 | 509 | 270 |
| 8 | 277 | 270 |
| 5 | 227 | 270 |
| 3 | 270 | 270 |
| 9 | 308 | 260 |
| 9 | 259 | 260 |
| 8 | 166 | 260 |
| 6 | 246 | 260 |
| 5 | 625 | 260 |
| 2 | 240 | 260 |
| 11 | 795 | 250 |
| 11 | 225 | 250 |
| 11 | 135 | 250 |
| 8 | 765 | 250 |
| 8 | 152 | 250 |
| 8 | 60 | 250 |
| 6 | 398 | 250 |
| 6 | 171 | 250 |
| 4 | 521 | 250 |
| 11 | 162 | 240 |
| 9 | 271 | 240 |
| 9 | 195 | 240 |
| 9 | 182 | 240 |
| 9 | 161 | 240 |
| 9 | 153 | 240 |
| 8 | 379 | 240 |
| 5 | 572 | 240 |
| 3 | 249 | 240 |
| 2 | 475 | 240 |
| 2 | 187 | 240 |
| 2 | 87 | 240 |
| 12 | 502 | 230 |
| 9 | 157 | 230 |
| 7 | 295 | 230 |
| 4 | 306 | 230 |
| 3 | 366 | 230 |
| 10 | 142 | 220 |
| 6 | 230 | 220 |
| 4 | 315 | 220 |
| 11 | 121 | 210 |
| 11 | 75 | 210 |
| 9 | 249 | 210 |
| 8 | 164 | 210 |
| 6 | 285 | 210 |
| 2 | 249 | 210 |
| 12 | 751 | 200 |
| 12 | 335 | 200 |
| 11 | 260 | 200 |
| 9 | 451 | 200 |
| 9 | 65 | 200 |
| 6 | 228 | 200 |
| 2 | 216 | 200 |
| 12 | 366 | 190 |
| 9 | 193 | 190 |
| 8 | 600 | 190 |
| 8 | 133 | 190 |
| 6 | 368 | 190 |
| 4 | 202 | 190 |
| 9 | 187 | 180 |
| 8 | 326 | 180 |
| 4 | 228 | 180 |
| 4 | 93 | 180 |
| 2 | 214 | 180 |
| 2 | 152 | 180 |
| 11 | 135 | 170 |
| 5 | 374 | 170 |
| 5 | 316 | 170 |
| 5 | 174 | 170 |
| 5 | 119 | 170 |
| 4 | 504 | 170 |
| 3 | 190 | 170 |
| 10 | 373 | 160 |
| 8 | 466 | 160 |
| 8 | 297 | 160 |
| 8 | 149 | 160 |
| 7 | 820 | 160 |
| 7 | 73 | 160 |
| 4 | 152 | 160 |
| 6 | 166 | 150 |
| 4 | 88 | 150 |
| 3 | 160 | 150 |
| 2 | 68 | 150 |
| 8 | 156 | 140 |
| 6 | 166 | 140 |
| 3 | 240 | 140 |
| 11 | 62 | 130 |
| 10 | 294 | 130 |
| 9 | 361 | 130 |
| 9 | 165 | 130 |
| 8 | 430 | 130 |
| 6 | 239 | 130 |
| 5 | 174 | 130 |
| 4 | 74 | 130 |
| 3 | 121 | 130 |
| 8 | 85 | 120 |
| 5 | 296 | 120 |
| 3 | 147 | 120 |
| 10 | 83 | 110 |
| 9 | 97 | 110 |
| 8 | 168 | 110 |
| 7 | 290 | 110 |
| 7 | 91 | 110 |
| 3 | 222 | 110 |
| 3 | 46 | 110 |
| 2 | 222 | 110 |
| 7 | 41 | 100 |
| 9 | 105 | 99 |
| 3 | 211 | 99 |
| 6 | 87 | 98 |
| 3 | 137 | 98 |
| 7 | 411 | 97 |
| 4 | 94 | 97 |
| 10 | 292 | 96 |
| 8 | 6 | 94 |
| 8 | 246 | 93 |
| 7 | 192 | 88 |
| 3 | 77 | 87 |
| 11 | 256 | 86 |
| 4 | 92 | 83 |
| 1 | 139 | 83 |
| 3 | 91 | 76 |
| 10 | 46 | 75 |
| 3 | 87 | 71 |
| 8 | 104 | 69 |
| 12 | 79 | 63 |
| 4 | 67 | 63 |
| 7 | 45 | 59 |
| 2 | 69 | 59 |
| 2 | 77 | 56 |
| 8 | 22 | 54 |
| 3 | 97 | 54 |
| 11 | 33 | 49 |
| 6 | 35 | 49 |
| 3 | 15 | 49 |
| 2 | 47 | 45 |
| 10 | 46 | 44 |
| 10 | 52 | 38 |
| 10 | 61 | 37 |
| 11 | 19 | 34 |
| 11 | 127 | 0 |
| 11 | 58 | 0 |
| 11 | 25 | 0 |
| 10 | 41 | 0 |
| 9 | 172 | 0 |
| 9 | 162 | 0 |
| 9 | 86 | 0 |
| 8 | 82 | 0 |
| 8 | 55 | 0 |
| 8 | 40 | 0 |
| 8 | 34 | 0 |
| 7 | 91 | 0 |
| 7 | 81 | 0 |
| 7 | 33 | 0 |
| 6 | 121 | 0 |
| 6 | 53 | 0 |
| 6 | 46 | 0 |
| 6 | 14 | 0 |
| 5 | 98 | 0 |
| 4 | 398 | 0 |
| 12 | 611 |  |
| 12 | 403 |  |
| 12 | 276 |  |
| 12 | 206 |  |
| 12 | 186 |  |
| 12 | 165 |  |
| 12 | 108 |  |
| 12 | 79 |  |
| 12 | 26 |  |
| 11 | 485 |  |
| 11 | 452 |  |
| 11 | 399 |  |
| 11 | 339 |  |
| 11 | 330 |  |
| 11 | 292 |  |
| 11 | 232 |  |
| 11 | 224 |  |
| 11 | 220 |  |
| 11 | 202 |  |
| 11 | 173 |  |
| 11 | 172 |  |
| 11 | 107 |  |
| 11 | 107 |  |
| 10 | 550 |  |
| 10 | 527 |  |
| 10 | 523 |  |
| 10 | 485 |  |
| 10 | 417 |  |
| 10 | 392 |  |
| 10 | 224 |  |
| 10 | 217 |  |
| 10 | 184 |  |
| 10 | 76 |  |
| 10 |  |  |
| 9 | 761 |  |
| 9 | 718 |  |
| 9 | 666 |  |
| 9 | 602 |  |
| 9 | 528 |  |
| 9 | 492 |  |
| 9 | 402 |  |
| 9 | 373 |  |
| 9 | 353 |  |
| 9 | 344 |  |
| 9 | 328 |  |
| 9 | 305 |  |
| 9 | 277 |  |
| 9 | 265 |  |
| 9 | 228 |  |
| 9 | 228 |  |
| 9 | 167 |  |
| 9 | 152 |  |
| 9 | 98 |  |
| 9 | 94 |  |
| 9 | 44 |  |
| 8 | 906 |  |
| 8 | 637 |  |
| 8 | 436 |  |
| 8 | 418 |  |
| 8 | 366 |  |
| 8 | 364 |  |
| 8 | 301 |  |
| 8 | 283 |  |
| 8 | 281 |  |
| 8 | 254 |  |
| 8 | 241 |  |
| 8 | 153 |  |
| 8 | 135 |  |
| 8 | 115 |  |
| 8 | 108 |  |
| 8 | 77 |  |
| 8 | 68 |  |
| 8 | 33 |  |
| 7 | 563 |  |
| 7 | 561 |  |
| 7 | 481 |  |
| 7 | 479 |  |
| 7 | 470 |  |
| 7 | 441 |  |
| 7 | 431 |  |
| 7 | 407 |  |
| 7 | 386 |  |
| 7 | 350 |  |
| 7 | 330 |  |
| 7 | 253 |  |
| 7 | 235 |  |
| 7 | 209 |  |
| 7 | 207 |  |
| 7 | 167 |  |
| 7 | 157 |  |
| 7 | 156 |  |
| 7 | 118 |  |
| 6 | 1148 |  |
| 6 | 830 |  |
| 6 | 673 |  |
| 6 | 644 |  |
| 6 | 385 |  |
| 6 | 381 |  |
| 6 | 244 |  |
| 6 | 230 |  |
| 6 | 226 |  |
| 6 | 221 |  |
| 6 | 199 |  |
| 6 | 186 |  |
| 6 | 118 |  |
| 6 | 106 |  |
| 6 | 84 |  |
| 6 | 74 |  |
| 5 | 748 |  |
| 5 | 698 |  |
| 5 | 519 |  |
| 5 | 498 |  |
| 5 | 480 |  |
| 5 | 476 |  |
| 5 | 465 |  |
| 5 | 369 |  |
| 5 | 354 |  |
| 5 | 336 |  |
| 5 | 325 |  |
| 5 | 283 |  |
| 5 | 279 |  |
| 5 | 245 |  |
| 5 | 125 |  |
| 5 | 83 |  |
| 5 | 56 |  |
| 5 |  |  |
| 4 | 682 |  |
| 4 | 662 |  |
| 4 | 572 |  |
| 4 | 517 |  |
| 4 | 471 |  |
| 4 | 432 |  |
| 4 | 391 |  |
| 4 | 377 |  |
| 4 | 377 |  |
| 4 | 306 |  |
| 4 | 256 |  |
| 4 | 253 |  |
| 4 | 225 |  |
| 4 | 224 |  |
| 4 | 211 |  |
| 4 | 207 |  |
| 4 | 200 |  |
| 4 | 195 |  |
| 4 | 178 |  |
| 4 | 170 |  |
| 4 | 158 |  |
| 4 | 114 |  |
| 4 | 93 |  |
| 4 | 89 |  |
| 4 | 73 |  |
| 4 | 45 |  |
| 4 | 45 |  |
| 4 | 33 |  |
| 4 | 32 |  |
| 4 |  |  |
| 3 | 705 |  |
| 3 | 664 |  |
| 3 | 437 |  |
| 3 | 337 |  |
| 3 | 325 |  |
| 3 | 307 |  |
| 3 | 302 |  |
| 3 | 290 |  |
| 3 | 287 |  |
| 3 | 234 |  |
| 3 | 229 |  |
| 3 | 213 |  |
| 3 | 212 |  |
| 3 | 199 |  |
| 3 | 195 |  |
| 3 | 181 |  |
| 3 | 167 |  |
| 3 | 157 |  |
| 3 | 156 |  |
| 3 | 149 |  |
| 3 | 143 |  |
| 3 | 105 |  |
| 3 | 71 |  |
| 3 | 58 |  |
| 3 | 48 |  |
| 3 | 34 |  |
| 2 | 382 |  |
| 2 | 331 |  |
| 2 | 313 |  |
| 2 | 283 |  |
| 2 | 273 |  |
| 2 | 271 |  |
| 2 | 213 |  |
| 2 | 209 |  |
| 2 | 183 |  |
| 2 | 180 |  |
| 2 | 169 |  |
